# SHEA 2022 Spring Proceedings without borders

**DOI:** 10.1017/ash.2022.247

**Published:** 2022-08-01

**Authors:** Alexandre R. Marra, Anucha Apisarnthanarak, Kari A. Simonsen, Kelly L. Matson, Pamela Bailey, Pranavi V. Sreeramoju, Priya Nori, Gonzalo M. Bearman

**Affiliations:** 1 Hospital Israelita Albert Einstein, São Paulo, Brazil; 2 University of Iowa, Iowa City, Iowa, United States; 3 Thammasat University Hospital, Pathumanthi, Thailand; 4 University of Nebraska Medical Center, Omaha, Nebraska, United States; 5 University of Rhode Island, Kingston, Rhode Island, United States; 6 University of South Carolina, Columbia, South Carolina, United States; 7 University of Texas Southwestern Medical Center, Dallas, Texas, United States; 8 Albert Einstein College of Medicine, Bronx, New York, United States; 9 Virginia Commonwealth University, Richmond, Virginia, United States

The SHEA 2022 Spring Conference provided a wide range of opportunities for networking with peers and experts in antimicrobial stewardship and healthcare epidemiology. It brought to light to a diversity of topics and innovative ideas unanswered or considered controversial in healthcare infection prevention. In the current times of global unrest related to the COVID-19 pandemic, climate change, war, and many other political, economic, and social adversities, high-quality science prevailed in the many SHEA abstracts. Even with the present-day healthcare challenges, overloaded by the daily work of caring for our patients, it was a joy to see professionals committed to presenting their data and their institutional or multi-institutional experiences in their scientific research.

We are pleased to present the SHEA 2022 Spring abstracts. One of the strengths of SHEA Spring Conference is its multidisciplinary approach to collaboration, which further increases the relevance of all research in the field of healthcare epidemiology. Participating in the conference’s scientific events is always a pleasure as well as an outstanding opportunity to share knowledge, discover different points of view, and collaborate with international researchers. Numerous SHEA 2022 Spring contributions were spread through the SHEA and ASHE Twitter accounts (@SHEA_Epi, @ASHE_Journal, @ICHEJournal), inspiring discussion and highlighting ideas and knowledge shared during poster and oral presentations.

Professorial rounds took place during breaks to discuss presentations on topics including antimicrobial stewardship, *Clostridioides difficile* infection, central-line–associated bloodstream infection, decolonization strategies, environmental cleaning, hand hygiene, infection control in low- and-middle-income countries, multidrug-resistant bacteria (eg, gram-negative rods, MRSA and VRE), pediatrics, surveillance and public health, respiratory virus, COVID-19, and much more. In total, 28 abstracts received an SHEA Abstract Award. These abstracts addressed fundamental topics within antimicrobial stewardship including diagnostic stewardship, β-lactam allergies, telestewardship, discharge stewardship, and outpatient parenteral antibiotic therapy (OPAT). They highlighted contributions to safer healthcare for outpatient antibiotic use via telehealth,^1^ educational perspectives to decrease antibiotic use for community-acquired pneumonia,^2^ quantifying risks for COVID-19 among nursing-home workers,^3^ resilient strategies developed by healthcare workers to improve compliance with COVID personal protective equipment,^4^ and discharging patients from the emergency room after drawing blood for culture in specific situations managed safely without compromising clinical outcomes.^5^

It is a privilege to acknowledge the hard work and dedication of so many professionals of diverse career levels. One of the selected top posters demonstrated that, despite lower overall antibiotic prescribing during the pandemic, data suggest that antibiotics, particularly azithromycin, are being prescribed for treatment of COVID-19 for which there is no demonstrable benefit.^6^ Another top poster showed that among hospitalized COVID-19 patients with advanced age and underlying comorbid conditions, coinfections were infrequent but were independently associated with increased mortality.^7^ This finding highlights the need for better tools to diagnose the presence or absence of bacterial and fungal coinfection in COVID-19 patients.

Another opportunity for researchers was the SHEA EPI Project Competition. The winning proposals were awarded up to $20,000 in grants to conduct studies that can further shape our understanding of the transmission of healthcare-associated infections and identify the best prevention methods and implementation science. Finalists presented their research concepts during the breakfast event. Selecting a winner among the finalists was a big challenge, and the Q&A focused on potential challenges researchers may face when implementing their research proposals. The 3 final proposals focused on (1) antimicrobial stewardship in home-care settings, (2) the integration of infection prevention and control into antimicrobial stewardship handshake rounds, and (3) the evaluation of the prevalence of multidrug-resistant bacteria (eg, carbapenemase-producing Enterobacterales) among patients admitted to acute-care hospitals from long-term care facilities. Each proposal is available on the SHEA YouTube channel. In reading the SHEA Spring 2022 abstracts, you will be immersed in real-world situations that ask and answer practical questions in the field of antimicrobial stewardship and healthcare epidemiology that contribute to the journey of safer patients and healthcare facilities. Finally, the SHEA Spring 2022 proceedings further the growth of *Antimicrobial Stewardship and Healthcare Epidemiology* as the home for the developing science of antimicrobial stewardship and infection prevention.
